# The Health Of Patients’ Eyes (HOPE) Glaucoma study. The effectiveness of a ‘glaucoma personal record’ for newly diagnosed glaucoma patients: study protocol for a randomised controlled trial

**DOI:** 10.1186/s13063-015-0863-2

**Published:** 2015-08-07

**Authors:** Marina Forbes, Helen Fairlamb, Leon Jonker

**Affiliations:** Clinical Nurse Specialist Ophthalmology, North Cumbria University Hospitals NHS Trust, Cumberland Infirmary, Ophthalmology Department, Newtown Road, Carlisle, CA2 7HY UK; Cumberland Infirmary, R&D Department, North Cumbria University Hospitals NHS Trust, Newtown Road, Carlisle, CA2 7HY UK

**Keywords:** Glaucoma, Ocular hypertension, Chronic open-angle glaucoma, Ophthalmology, Patient education, Knowledge, ‘Glaucoma personal record’, Client-held eye summary, Individual record of care, Randomised trial

## Abstract

**Background:**

Glaucoma involves progressive optic nerve fibre loss, subsequently leading to irreversible and disabling visual field defects. In Europe, the prevalence of glaucoma is approximately 2.2 % of all people aged over 40 years; this equates to 12 million people. Glaucoma patients require regular lifelong follow-up, contributing to a large financial and resource burden for the National Health Service (NHS) in the UK. This study aims to determine whether providing newly diagnosed glaucoma patients with a personalised client-held eye health summary (‘glaucoma personal record’), improves patients’ knowledge of their glaucoma condition. A potential long-term benefit could be improved self-management and henceforth a slower rate of deterioration.

**Methods/Design:**

HOPE Glaucoma is a 3-year, prospective, parallel-group, pragmatic, single-centred, randomised controlled trial. An anticipated 122 adults, newly diagnosed with glaucoma (including ocular hypertension, suspected glaucoma and/or chronic open-angle glaucoma) will be recruited from a nurse-led ophthalmology outpatient clinic at a medium-sized NHS Trust. Participants will be randomly allocated to receive standard clinical care (control arm) or standard care plus a glaucoma personal record, detailing the current state of their condition (interventional arm). Participant assessments are designed to test whether provision of a glaucoma personal record 1) improves patient knowledge of glaucoma and 2) contributes to improvements in clinical outcomes, i.e. delay of visual field loss. The primary outcome measure is better client knowledge of glaucoma at the 9–12 month follow-up visit. Secondary outcome measures include the rate of visual field loss and patient-reported outcome measures on visual function (National Eye Institute VFQ – 25) measured at baseline, 9–12 months, 24 months and 36 months. Estimating a 20 % drop-out rate, the study will have 90 % power to detect a mean two-point difference in glaucoma knowledge score between groups at 5 % significance - based on two-sided Mann–Whitney *U* test.

**Discussion:**

If a glaucoma personal record is found to significantly improve glaucoma patients’ knowledge of their condition, this intervention could potentially provide a low-cost, straightforward tool to educate and engage glaucoma patients. Subsequently, this could have the potential to increase patient self-management and therefore allow glaucoma patients to prolong their sight functionality for longer.

**Trial registration:**

ISRCTN41306818, registered on 22 August 2013.

**Electronic supplementary material:**

The online version of this article (doi:10.1186/s13063-015-0863-2) contains supplementary material, which is available to authorized users.

## Background

Glaucoma encapsulates a disparate group of eye diseases that are multi-factorial and individual to each patient. The common factor is progressive optic nerve fibre loss, which leads to irreversible and disabling visual field defects if it is not diagnosed and treated early. It is estimated that in Europe approximately 12 million people are affected by glaucoma, equating to 2.2 % of the population aged over 40 years [1]. These individuals require lifelong review to monitor their condition and response to topical ocular antihypertensive eye drops and/or other treatments. As the population ages, glaucoma will continue to present a significant burden for our current healthcare resources [2]. Improved knowledge and understanding has the potential to enhance compliance and any tool which increases patients’ understanding of their glaucoma should be beneficial to all stakeholders. Beneficiaries of this research are presented in Table 1.

**Table 1:** Beneficiaries of increased client understanding of their glaucoma

Beneficiaries	Reasons
Patients/clients	Receive appropriate knowledge, support and motivation to manage their condition to remain sighted for their lifetime.
Family and caregivers	Glaucoma can be hereditary. Significant life-changing effects to family and/or caregivers if visual loss is not prevented. If relative remains sighted for their lifetime, burden of care is reduced.
Medical and nursing staff in acute care	Clients carry an individualised summary of the current state of *their* glaucoma thus increasing communication and continuity of care.
Ophthalmic department and organisation (the Acute Trust)	Increased capacity to care for engaged glaucoma clients, resulting from good resource use and high-quality service.
General practitioners and community optometrists (primary care)	Glaucoma clients successfully self-manage their care, and can share their eye summary and health state with primary care healthcare professionals.
Local healthcare economy	Clients diagnosed with a long-term sight-threatening condition are managed within current resources, and the burden of visual disability within the community is curbed.
Clinical nurse glaucoma specialist	Job satisfaction. Professional kudos. Sense of value to all those above, but especially clients. Enjoy sharing knowledge and skills and supporting client group.

There are various client-held care records utilised throughout the UK healthcare services [3–5], yet there is a paucity of such records specifically designed for those with glaucoma. Previous research has recognised that clients who receive emotional support and information at the initial diagnosis of glaucoma benefit by displaying good compliance and co-operation with their care [6]. Individualised care of glaucoma patients, taking into account healthcare needs and patient beliefs about illness and medicines, has also been shown to improve adherence to ocular hypotensive therapy and have the potential to delay progression of the condition [7].

There is currently no available evidence of previous efforts to produce a client-held ‘glaucoma personal record’ for secondary care ophthalmic units and the authors are not aware of any research assessing whether client-held glaucoma personal records produce better health outcomes in glaucoma [8]. Clinical teams may provide numerous resources to clients, depending on the individuals’ specific needs, yet there is potential to produce a standardised summary that can be tailored to record each individual’s current appearance of their glaucoma status. This summary should add value to client care as it would be held by each individual and personalised to reflect *their* condition. One would expect that providing glaucoma clients with their own personal ‘glaucoma personal record’ or client-held eye summary, at diagnosis, may potentially help to engage them more effectively with their diagnosis and increase their understanding.

National Institute of Clinical Excellence (NICE) guidelines on the diagnosis and management of chronic open-angle glaucoma and ocular hypertension recommend that research is required to assess “what is the clinical effectiveness and cost effectiveness of providing people with ocular hypertension (OHT), suspected glaucoma (SG) and/or chronic open-angle glaucoma (COAG) with a ‘glaucoma personal record’ compared with standard treatment?” [8]. This study aims to fulfil this requirement by assessing the clinical effectiveness of providing patients with OHT, SG and/or COAG (henceforth collectively referred to as glaucoma) with an in-house developed ‘glaucoma personal record’, when compared with current standard best practice.

Clinical effectiveness will primarily be measured by assessing patient knowledge concerning his/her own condition at 9–12 months, to coincide with a regular follow-up visit in clinic. Furthermore, client perception of how their glaucoma affects daily living and ophthalmological clinical parameters will be measured annually for 3 years. The ultimate aim is to instigate methods of enhancing self-management through increased glaucoma awareness and potentially achieving deceleration of glaucoma progression in patients.

## Methods

### Trial design

This 3-year, prospective, single-centre, parallel-group, randomised, controlled clinical trial will assess the effect of graphic eye-health information provision to patients with glaucoma or risk of glaucoma.

### Participants and settings

Newly referred patients attending a nurse-led ophthalmology clinic in a medium-sized hospital in the North of England will be assessed by the chief investigator (CI), an ophthalmologic nurse specialist, for suitability using the criteria detailed below:

### Inclusion criteria

- Adult (age ≥18 years)
- Patients newly diagnosed with glaucoma, to include the following three presentations:

- o ocular hypertension (OHT): intraocular pressures (IOPs) above the normal range (more than or equal to 21 mmHg), but no signs of optic nerve fibre loss or visual field loss
- o suspected glaucoma (SG): optic nerve fibre loss or visual field loss or both in presence of normal intraocular pressures
- o chronic open-angle glaucoma (COAG): any combination of raised intraocular pressures, optic nerve fibre loss and/or visual field loss.
- o any combination of the above OHT, SG, COAG

- Both primary (cause of outflow resistance or angle closure is unknown) and secondary (outflow resistance results from another disorder) glaucoma cases can be included such as pigment dispersion syndrome (PDS) or pseudoexfoliative glaucoma (PEXF)

### Exclusion criteria

- Under age (<18 years)
- Mental incapacity through inability to read, co-morbidities (e.g*.* severe stroke, advanced dementia) or any other contributing factor
- Inability to provide informed written consent
- Unable to speak or understand English since an interpreter is not available throughout the course of the study.
- Previous diagnosis of glaucoma

Other co-morbid ophthalmological conditions, such as cataracts and age-related macular degeneration are not listed as exclusion criteria to ensure that the study is pragmatic.

Patients meeting the eligibility criteria will be recruited into the trial by the CI from a ‘new glaucoma’ referral clinic. Recruiting newly diagnosed adults, with little or no experience of previous glaucoma services, will reduce prior experience of secondary ophthalmic care and potential educational bias.

### Consent

If eligible, patients will be given verbal and written information on the study by the CI during their consultation to avoid sensitisation to the objectives of the study. One key aspect of the study is to avoid priming patients about the potential benefit of education (utilising a post-test-only design). If patients are willing to participate, written informed consent will be obtained by the CI at the same consultation. There is, therefore, a limited time frame for patients to make a decision about participation. However, the patient information sheet will stress that there is a ‘cooling-off’ period and that patients can change their mind regarding participation at any given time. If a participant is already enrolled in another study, then he/she will be allowed to participate in this trial provided the other study does not exclude him/her from doing so.

### Sample size

The anticipated patient sample size of 122 will be recruited over a 12-month period. Power calculations for sample size, 90 % power and 5 % significance, based on two-sided Mann–Whitney *U* test for the primary outcome measure, patient knowledge of glaucoma. A priori power calculations, using GPower 3.1 software, result in the following sample sizes summarized in Table 2. The calculation also takes into account a 20 % drop-out rate over the initial 9–12-month follow-up period before patients return for their regular glaucoma follow-up and knowledge questionnaire visit.

**Table 2:** Sample size calculation

Mean score control group (± SD)	Mean score intervention group (± SD)	Required total sample size, n
7 (3)	8 (3)	400 (200 per group)
7 (3)	9 (3)	122 (61 per group)
7 (3)	10 (3)	58 (29 per group)

*SD* standard deviation

### Randomisation

**Fig. 1:**
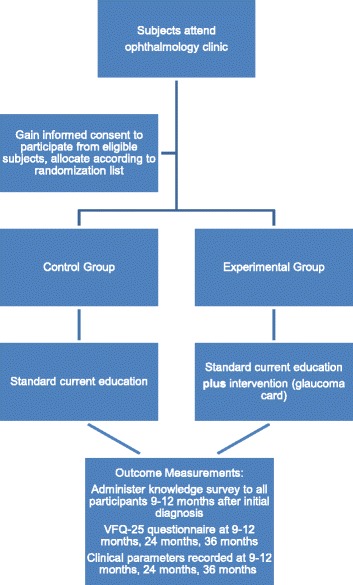
HOPE trial schema

Each participant will be assigned to the control or interventional arm by a pragmatic randomisation approach; there will be no stratification and randomisation will take place on a 1:1 ratio without blocking. Online freeware will be used to generate a random allocation sequence in advance of the recruitment period, seen only by the trial administrator (who will not be involved with data collection) [9]. Allocation concealment will be achieved using consecutively numbered, sealed, opaque envelopes prepared in advance of recruitment by the trial administrator. This will ensure the investigator is not aware of the allocation sequence in advance of randomization. Eligible subjects will be randomised and - if assigned to the intervention arm - presented with their glaucoma personal record by the CI immediately after written informed consent has been obtained. For overview of the study process, see Fig. 1.

### Intervention description

Both groups will have exposure to current educational resources and materials presented at diagnosis, as is standard clinical practice. This may include an enlarged model of the eye, ocular anatomy posters, ocular coherence tomography sample scans, images of healthy and damaged optic nerve heads, examples of normal visual field tests and those showing glaucomatous loss, and a glaucoma information leaflet to take away with them. The chief investigator’s contact number will also be provided, as would be standard in her capacity as clinical nurse specialist in ophthalmology.

Those participants allocated to the interventional arm will, in addition to standard clinical care, be provided with their own ‘glaucoma personal record’ (Additional file 1), which will be personalised to document the current state of each participants’ glaucoma diagnosis and utilised during ophthalmologic review. Both groups of participants will be asked to complete the National Eye Institute Visual Functioning Questionnaire – 25 (VFQ-25) to gain an insight into patients’ perceptions about the impact that glaucoma may have on their daily living. The ‘glaucoma personal record’ will be administered to those participants randomised to receive the intervention by the CI immediately after the informed consent process has been completed.

### Intervention development

Recent work by Waterman [10] identified the need for patients to be informed about their glaucoma, to help them understand their condition and the implications of poor medication adherence. The glaucoma personal record used in the HOPE Glaucoma trial (Additional file 1) was developed from the concept of the Spaeth-coloured glaucoma graph for diagnosed glaucoma patients and glaucoma suspects [11]. This graph provides both patients and clinicians with visual information concerning current optic disc appearance. Spaeth and Paulus [11] claimed it is user-friendly because of the traffic light system. They also claimed that most patients understand that green indicates ‘safe’, yellow/amber means ‘caution’, and red means ‘danger’. They proposed that “the graph can help in patient counselling by increasing the understanding regarding the disease status”. The work from Spaeth and Paulus inspired application of a traffic light system to visual field scoring using the Hoddap Classification [12, 13].

Although staging systems may be of limited value in clinical practice, the authors intended to put together an inexpensive yet useful ‘glaucoma personal record’. The Hoddap system has three stages, namely, early, moderate and advanced glaucomatous loss. The rate of progression of visual field loss will be measured by regression analysis of the mean deviation from the mean, expressed in decibels per year. Since this glaucoma personal record is intended to help patients understand their current measures according to a traffic light system, best evidence available has to be balanced with simplicity, in order to be useful.

The third fundamental measure obtained at regular glaucoma review appointments is intraocular pressure (IOP). Elevated IOP above 21 mmHg is considered a major risk factor in glaucoma pathology. Normal IOP is considered to be less than or equal to 20 mmHg. Applying the traffic light system here means lower IOPs lie within the green ‘safe’ zone. IOPs between 21 and 30 mmHg - the amber zone - are observed with caution, although many other factors must be taken into consideration e.g. central corneal thickness, visual field loss or suspicious optic discs. Any IOP above 30 mmHg, the red zone, usually means antihypertensive treatment is required as risk of glaucomatous damage or vein occlusion is considered too high to leave untreated.

The current glaucoma personal record under investigation is a coloured, A5-sized (or A4 if required), booklet of 16 pages. It was refined with advice from the International Glaucoma Association, who provided financial sponsorship for this study. This prototype glaucoma personal record is open to development and further refinement, depending on feedback and outcomes of this study.

### Objective

This study aims to assess whether patient education, through the provision of a glaucoma personal record, results in increased knowledge of their condition and whether this contributes to positive behavioural change and a reduction in the rate of visual field loss. The null hypothesis is that a patient-specific glaucoma personal record has no effect on client knowledge of glaucoma (OHT, SG or CAOG), quantified using a validated researcher-conducted survey at 9–12 months post-recruitment.

### Outcome measures

#### Primary outcome

The primary outcome measure is client knowledge of glaucoma, quantified using a validated researcher-conducted survey at 6–12 months post-recruitment [8], see Table 3. This survey and scoring system was used, and validated by ophthalmic colleagues in 2009/2010 “to establish the impact of educational support on clients’ knowledge of glaucoma and adherence, in preparation for a further interventional study” [9]. It provides an ordinal score of participants’ knowledge. The primary outcome measure is therefore the degree of understanding and knowledge retention of their OHT, SG, or COAG in the interventional group compared with the control group. Patients requiring ocular antihypertensive treatment at first presentation are eligible for inclusion in this trial; the total knowledge score achievable is either 12 for those not prescribed antihypertensive drops, or 17 for those prescribed eye drops during the course of the study. The survey will be administered by the CI when participants attend a ‘glaucoma review’ outpatient clinic between 6 and 12 months post-recruitment.

**Table 4:** Overview of study timeline and outcome measures

Baseline	Baseline	9–12 months	24 months	36 months
Patient knowledge		X		
Patient perspective (VFQ-25)	X	X	X	X
Clinical parameters	X	X	X	X

*VFQ-25* National Eye Institute Visual Functioning Questionnaire – 25

#### Secondary outcomes

The secondary, longer-term, outcome measure is rate of visual field loss. In line with clinical practice, visual field loss will be measured at least annually (more frequently if there is a clinical indication). The relative visual loss rates between control and intervention groups will also be measured annually. Visual field testing will be performed by standard automated perimetry (SAP) using the Humphrey Central 24–2 programme. The Humphrey perimeter provides print-outs with statistical analyses of single field-test results, including numerical indices of reliability, and deviation from the norm of age-matched visual field analyses. The visual field indices used to identify progression of visual field loss will be mean deviation (MD) from the mean. MD represents the average difference between normal age-corrected sensitivity values and the subjects’ measured values at all test points. The rate of progression of visual field loss will be measured by linear regression analysis of the MD from the mean, expressed in decibels per year (or the newer visual field index, which was not available at the start of this study).

A simple measure was selected for use in this trial. However, there are challenges to using visual field analyses to detect progression. The test is subject to variability in performance, even for the same patient. Modern perimetry attempts to reduce sources of error in, for example, reduced test time, continuous gaze/fixation monitoring. The role of the operator is crucial in assisting patients to perform the test as accurately as possible [13]. All visual field testing will be undertaken by the CI to reduce inter-assessor variability.

Trend analyses to quantify rate of visual field loss require at least five tests to detect progression. This means relatively frequent testing in the first 2 years following diagnosis, especially if patients initially present with loss. The European Glaucoma Society (EGS) suggest three tests a year in the first 2 years. As such, this secondary outcome measure will take approximately 2–5 years to establish. The MD score will be documented in the ‘glaucoma personal record’ for the intervention group, and obtained from standard medical records for the control group.

The impact of visual field loss on patients’ lives will be measured using the vision-targeted health-related quality of life (HRQOL) measure: the National Eye Institute Visual Functioning Questionnaire – 25 (VFQ-25). This validated patient-reported outcome measure tool was designed to measure the impact of visual deficit on physical functioning, emotional well-being and social functioning [14]. The VFQ-25 will be completed by participants attending the CI’s nurse-led glaucoma review outpatient clinic at baseline, 6–12 months, 24 month and 36 months post-recruitment.

The patient glaucoma record will use ‘traffic light’ graphs to make it more straightforward for patients to see the status of their glaucoma for e.g. visual field loss [11]. A copy of the patient glaucoma record is available (Additional file 1).

#### Tertiary outcome

Participants’ demographic data will be collected and analysed to compare the distribution between control and intervention groups in terms of: age, gender, postcode and highest level of education achieved. The latter two are of particular relevance as the study will assess self-education and disease awareness [15]. The control and intervention groups will also be stratified by disease diagnosis (OHT, SG and COAG), although the aforementioned primary and secondary outcome measures are based on collective data.

Other potential confounding factors that will be recorded for each participant are related to general eye health and the resulting management of the newly diagnosed glaucoma. It will be recorded if patients: have never worn glasses/lenses, wear glasses/lenses due to old age (reading and/or driving), wear glasses since childhood or early adulthood due to congenital visual defect. Note will also made whether patients are prescribed IOP-lowering eye drops or undergo a laser or surgical intervention during the study period.

### Data collection and monitoring

Participant follow-up will be 3 years. The estimated primary completion date is September 2015 and the estimated study completion date is September 2017. An overview of the study timeline and recorded outcome measures are detailed in Table 4. Outcome data will be collected during clinically scheduled ophthalmology outpatient appointments with the CI and subjects will not be asked to attend any additional clinic appointments above those required for their clinical care. This design was used to increase the pragmatism of the study and improve subject retention throughout the duration of the study. Outcome data will not be collected for patients that withdraw from the study or deviate from the study timeline.

**Table 3:** The scoring system used to establish participants’ knowledge of glaucoma and its management. Source: [9]

Number	Question	Scored 0	Scored 1	Scored 2	Scored 3	Max score
1	Can you tell me what you think glaucoma is?	Don’t know/ incorrect answer	Build up of pressure, raised/high pressure, nerve damage, loss of vision	Optic nerve damage, irreversible vision loss, chronic disease, slowly progressive (in own words)	NA	2
2	Can you tell me which part of the eye can become damaged with glaucoma?	Don’t know/ incorrect answer	Back of the eye	Retina	Optic nerve	3
3	Can you tell me what effect glaucoma has on vision if left untreated?	Don’t know/ incorrect answer	You go blind/you gradually go blind/ you lose vision	You get tunnel vision	NA	2
4	Can you tell me what part of vision glaucoma affects the most?	Don’t know/ incorrect answer	Visual field/field of vision/side vision	NA	NA	1
5	Is glaucoma hereditary/does glaucoma run in families?	No/don’t know	Yes	NA	NA	1
6	Do you know what investigations/tests will be carried out regularly at future clinic appointments to monitor changes in your eyes?	Don’t know/ incorrect answer	Either IOP measurement, visual field test or optic nerve examination (in own words)	Any two of the tests (in own words)	All three tests (in own words)	3
7	Do you know the name(s) of the drop(s) that has/have been prescribed for you?	Don’t know/ incorrect answer	Yes (client named the drops)	NA	NA	1
8	Do you know what the drops do?	Don’t know/ incorrect answer	Lower/reduce/control eye pressure	NA	NA	1
9	Do you know when you will need to collect your next prescription?	Don’t know/ incorrect answer	1 month/28 days	NA	NA	1
10	After opening a bottle of drops do you know how long you can use them before they reach their expiry date?	Don’t know/ incorrect answer	1 month/28 days	NA	NA	1
11	Do you know how long you will have to use drops for?	Don’t know/incorrect answer	For life/forever	NA	NA	1

Maximum total score achievable: 17. *IOP* intraocular pressure

All data will be entered into a trial-specific database by a single administrator as they accumulate, access to which will be restricted and secure. All trial data will be pseudoanonymised by use of unique participant trial numbers. Access to the final data set will be limited to the trial administrator (HF) and statistician (LJ). A data monitoring committee will not be utilised throughout the study, but the study sponsor will audit study conduct and data quality as part of the sponsors’ existing annual audit process. As the study intervention has no impact on the clinical management of glaucoma patients, there are minimal safety concerns for those involved with the HOPE Glaucoma study. Data on solicited and spontaneously reported adverse events will not be collected.

### Data analysis

Patient eligibility, recruitment and retention through the study will be presented in a CONSORT flow diagram displaying the number of eligible patients, patients approached with study information, patients recruited and randomised and patients completing study follow-up. Reasons for loss to follow-up will also be presented, where known. Participant baseline details and descriptive statistical data will be tabulated to show overall recruit demographics, and any differences in distribution (e.g. patient age, sex, level of education) will be analysed by applying one-way ANOVA or chi-squared test.

The primary outcome, the degree of difference in patient knowledge about their glaucoma condition will be analysed by comparing the average scores for control patients versus the average score of patients received the glaucoma personal record. Statistical significance will be assessed by applying the Mann–Whitney *U* test. The secondary outcome, visual field loss, will be measured with a Humphrey Automated Visual Field Analyser in the ophthalmology clinic and average measurements for baseline visit and the final visit at 36 months will be compared within each group (control and intervention respectively) and also between the two groups. Depending on distribution of the data, determined by Shapiro-Wilk test, the paired *t* test or Wilcoxon test will be applied for paired samples to determine any significant changes in visual field loss. For assessing differences between the control and intervention groups, either *t* test or Mann–Whitney *U* test will be applied. Like visual field loss, the other secondary outcome measure - patient-related outcome measure VFQ-25 - will be presented as average scores. Any statistical differences will be assessed by Wilcoxon test within each group and Mann–Whitney *U* test between groups.

### Research governance

Approval and support for this study has been obtained from the following bodies: the National Research Ethics Service (reference 12-YH-0471) and the sponsor, North Cumbria University Hospitals NHS Trust. Contact person for the sponsor is the R&D director, Dr Jim George. There is no provision made for non-negligent liability; the CI and other research team members are covered by standard NHS indemnity. The study is funded by a Nurse Research Grant from the International Glaucoma Association, based on the ethically approved study protocol (version 1, 19 September 2012) and supporting study documents (i.e. patient information sheet, consent form, ‘glaucoma personal record’) [16].

### Publication and data-sharing policy

The study results will be presented at (inter)national scientific meetings and at local seminars and conferences. The results from the study will be written up and submitted to a peer-reviewed journal. We intend to write a summary sheet for distribution in, e.g., ophthalmology waiting areas. This summary sheet can also be distributed to participants upon request. All published data will not contain information that can identify any of the subjects who have participated in the study, in line with the Data Protection Act [17]. Each participant will be given a study number so that the data can be pseudoanonymised.

## Discussion

The NICE Guideline Development Group published recommendations for research into the clinical effectiveness of providing people with COAG with a ‘glaucoma personal record’ when compared with standard treatment [8]. To the authors’ knowledge, the HOPE Glaucoma study constitutes the first effort towards fulfilling NICE recommendations and the planned publication of study findings in circa 2017 will provide the first data generated by a randomised controlled trial on ‘glaucoma personal record’ provision. Findings will strengthen the evidence base available for glaucoma management guideline development and may have a positive impact upon the future education and care of glaucoma patients.

Strengths of the trial include the use of a time-matched control, the random allocation of the ‘glaucoma personal record’, allocation concealment to minimise selection bias, long-term recruit follow-up (36 months) and the use of validated questionnaires to capture outcome data. Due to its fairly pragmatic approach, the trial is not without limitations. The decision not to collect detailed data on patient co-morbidities could omit potential confounding factors throughout this trial. Other foreseeable study limitations include a limited patient demographic, due to recruitment from a single-centre in a rural location with a limited ethnic variety. The generalisability of trial findings may also be limited by the selection of participants from a nurse-led clinical nurse specialist new glaucoma referral clinic only. Gray et al*.* [9] indeed found that the depth of information provided for newly diagnosed patients varied across clinics and between individual clinicians. Despite this limitation, the use of one glaucoma practitioner throughout the duration of the trial offers a means of standardising the type and quality of information and materials given to all participants at diagnosis and during follow-up review. Variability in the nature of the information provided at diagnosis and in the quality of outcome measure assessment will be minimised, as the CI will be responsible for the provision and adaptation of care for all participants, according to the individual clinical need, and regardless of the arm to which they were allocated.

One other limitation of the trial includes the risk of bias. Due to the nature of the intervention - provision of a booklet - it is not feasible to mask participants to which arm of the study they will be allocated to. The CI is responsible for determining the suitability of participants, allocating the intervention and collecting data for the outcome measures in this trial, which introduces other sources of potential bias. The outcome assessor will not be masked to the interventional group, as the assessor distributed the ‘personal glaucoma record’ and clients will return with (or refer too) their record at follow-up; this increases the risk of detection bias. However, the utilisation of a second outcome assessor was deemed unworkable, and a pragmatic approach with one assessor was adopted.

The use of a ‘glaucoma personal record’, such as the one produced for this trial, for those diagnosed with OHT, SG and/or COAG may provide an additional, low-cost source of information to patients and other ophthalmic healthcare professionals which is easily produced and administered. Glaucoma personal records may have the potential to improve patients’ understanding of their condition, help reduce patient uncertainty and potentially improve medication adherence [18].

## Trial status

Recruitment to the HOPE Glaucoma study began in June 2013. Recruitment is ongoing, with 85 % of the sample size achieved. The study is anticipated to reach the recruitment target of 122 participants in September 2014.

## Additional file

Additional file 1:
**Glaucoma personal record.** This is the study intervention;a booklet that can be personalised to contain participant’s clinical information relevant to their glaucoma. Only participants randomised to the interventional arm are provided with this booklet. (PDF 320 kb)

## Abbreviations

CI: chief investigator
COAG: chronic open-angle glaucoma
HRQOL: health-related quality of life
IOP: intraocular pressure
MD: mean deviation
OHT: ocular hypertension
PDS: pigment dispersion syndrome
PEXF: pseudoexfoliative glaucoma
SAP: standard automated perimetry
SG: suspected glaucoma
VFQ-25: National Eye Institute Visual Functioning Questionnaire – 25
