# Supplementary material for: The Health Of Patients’ Eyes (HOPE) Glaucoma study. The effectiveness of a ‘glaucoma personal record’ for newly diagnosed glaucoma patients: study protocol for a randomised controlled trial
Source: Trials. 2015 Aug 7;16:337. doi: 10.1186/s13063-015-0863-2 (PMC4528378; doi:10.1186/s13063-015-0863-2)
Supplement: Additional file 1: — Glaucoma personal record. This is the study intervention;a booklet that can be personalised to contain participant’s clinical information relevant to their glaucoma. Only participants randomised to the interventional arm are provided with this booklet. (PDF 320 kb) [file 13063_2015_863_MOESM1_ESM.pdf]

North Cumbria University Hospitals NHS Trust

HOPE Glaucoma study

Health Of Patients' Eyes (HOPE)

Stick patient label here

Contact:

Sister Marina Forbes

Clinical Nurse Specialist

Ophthalmology

Tel: 01228 814323

Patient card v5 dd 7 May 2013

Your current diagnosis

	Right eye	Left eye
Glaucoma suspect		
Open Angle Glaucoma		
Narrow Angle Glaucoma		
Ocular hypertension		
Suspicious discs		
Other:		

Your glaucoma booklet

This booklet contains information specifically about **your glaucoma** (for availability of more general information, see the back page, page 16).

Different figures and graphs are used to show the status of your glaucoma, and if it is getting worse or not. The colours used in mean the following:

Red - your current measures indicate advanced damage

Amber - your current measures mean we need to be vigilant

Green - your current measures give us little concern

Your optic discs appearance

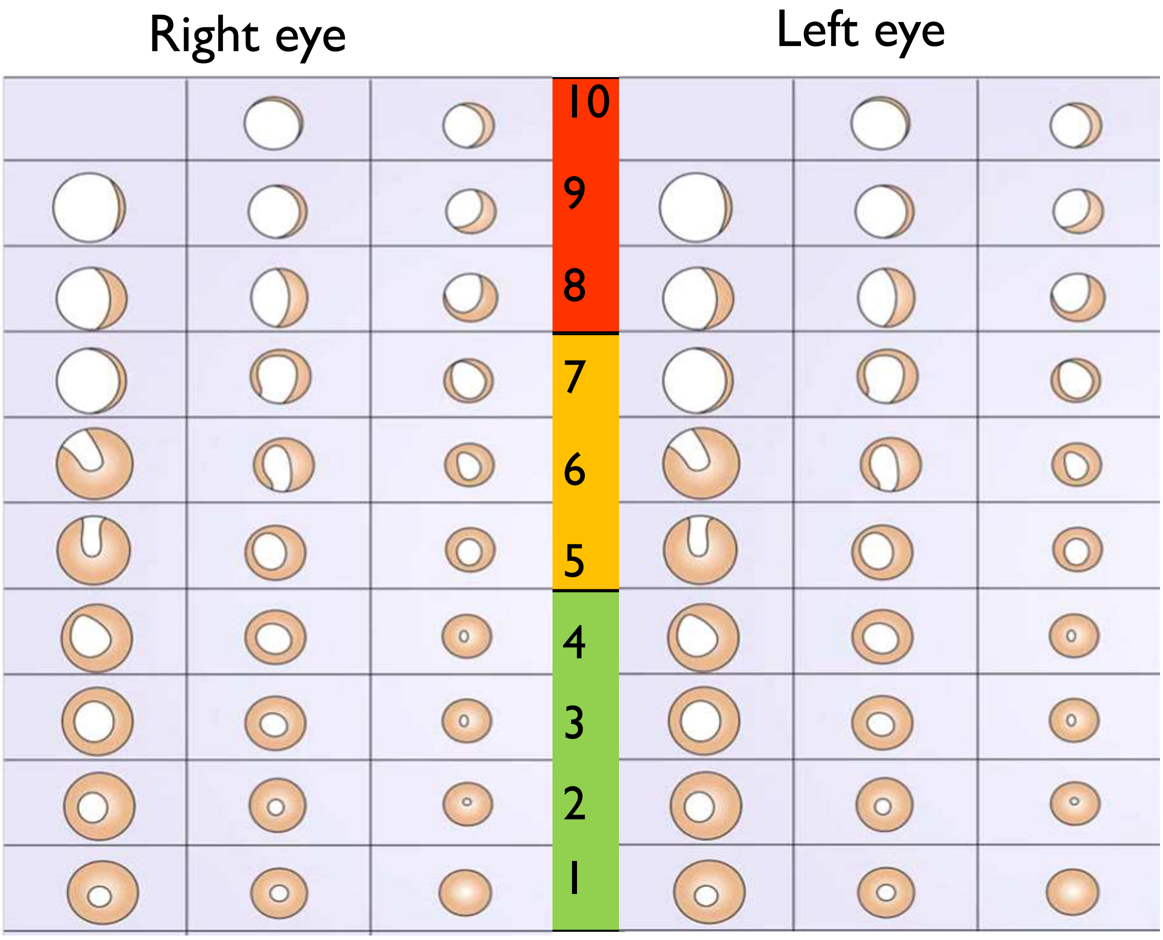

What does it mean: the Disc Damage Likelihood Scale rates how your optic nerve disc appears to us on examination. Optic nerve heads can differ in size, that is why three sizes are shown (small, average, large).

The more space (or ‘cupping’, shown in white) there is inside the optic disc, the higher and worse (higher) the score.

Your disc damage, Right Eye

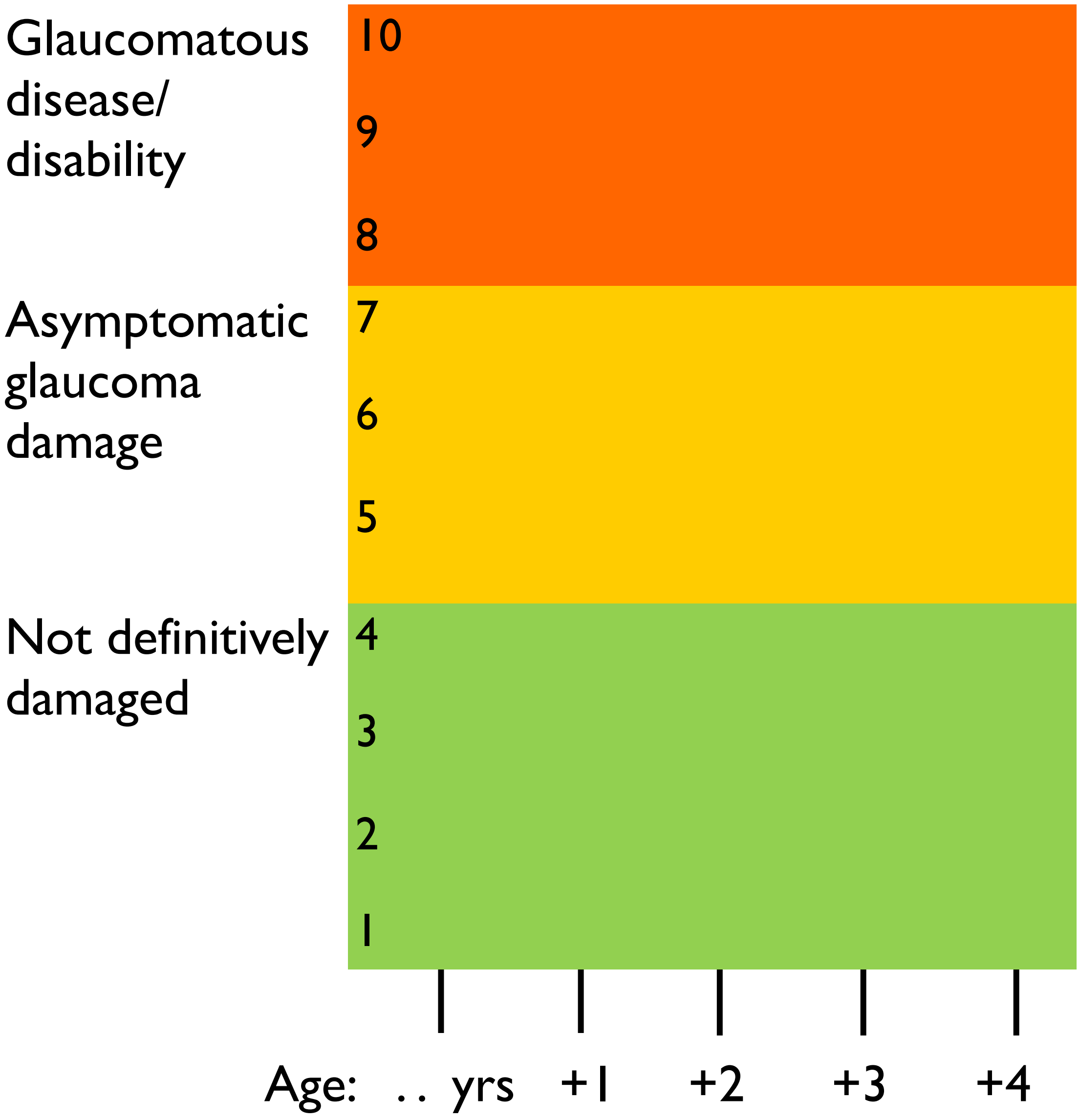

What does it mean: the Disc Damage Likelihood Scale rates how your optic nerve heads (discs) appear to us on examination. The scoring here on page 4 and 5 uses the Disc Damage Likelihood Scale, just like on page 3.

Your disc damage, Left Eye

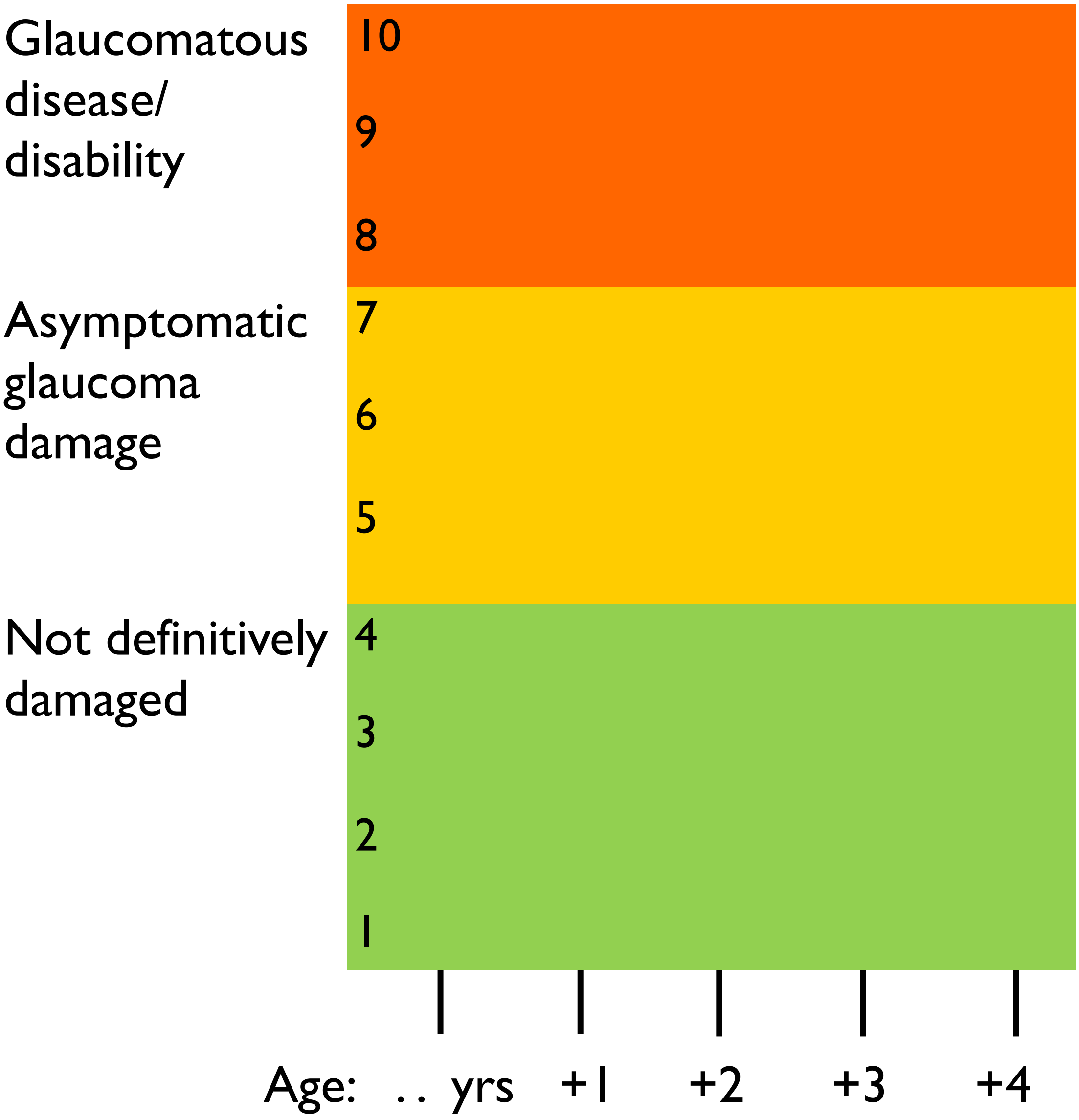

Your visual field score, Right Eye

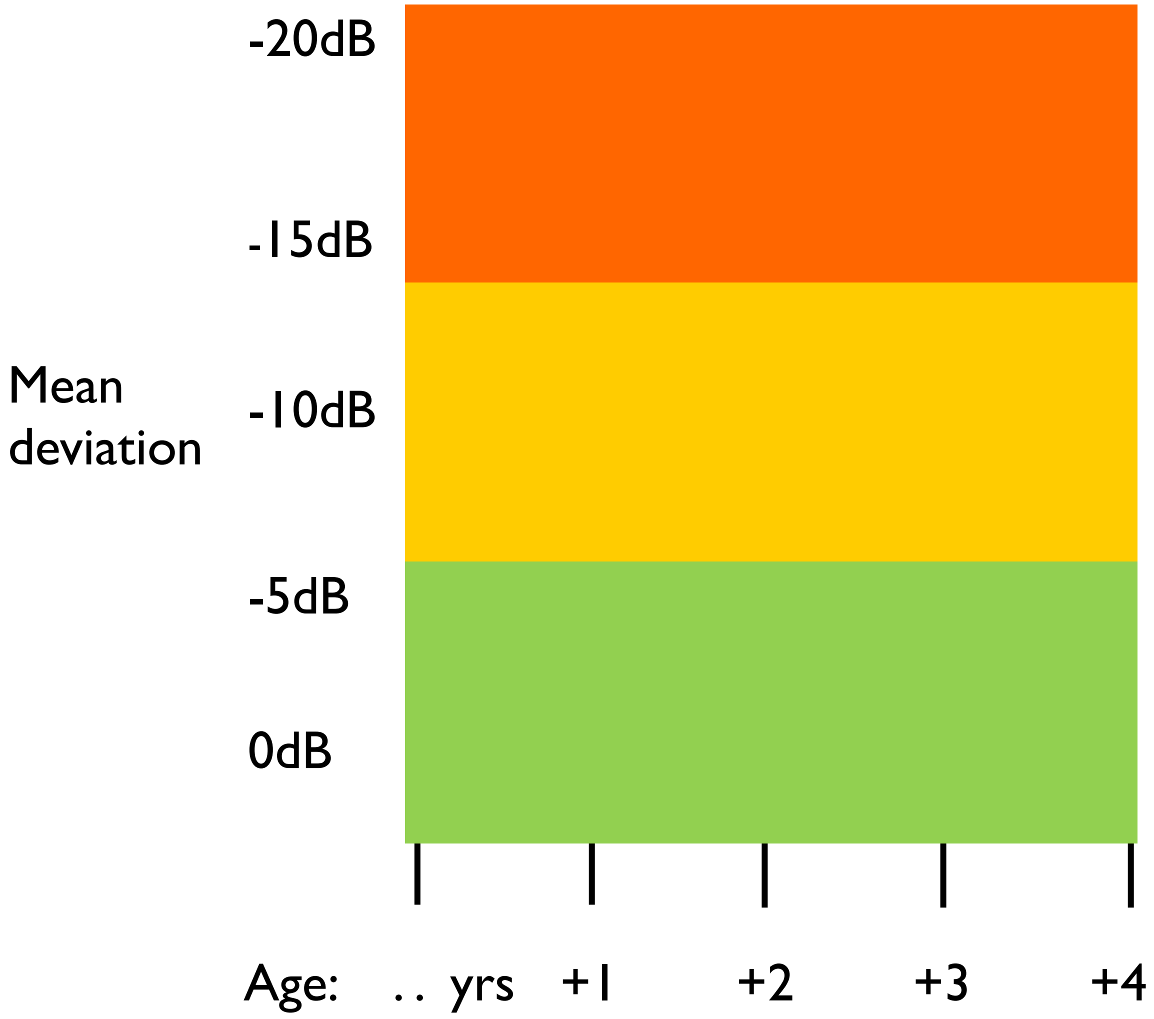

What does it mean: The field of visual test measures your central and peripheral vision. The mean deviation shows how well you performed. A zero (0 dB) means you have the same score as other patients in the same age group as you. A negative number means worse than other patients in the same age group.

Your visual field score, Left Eye

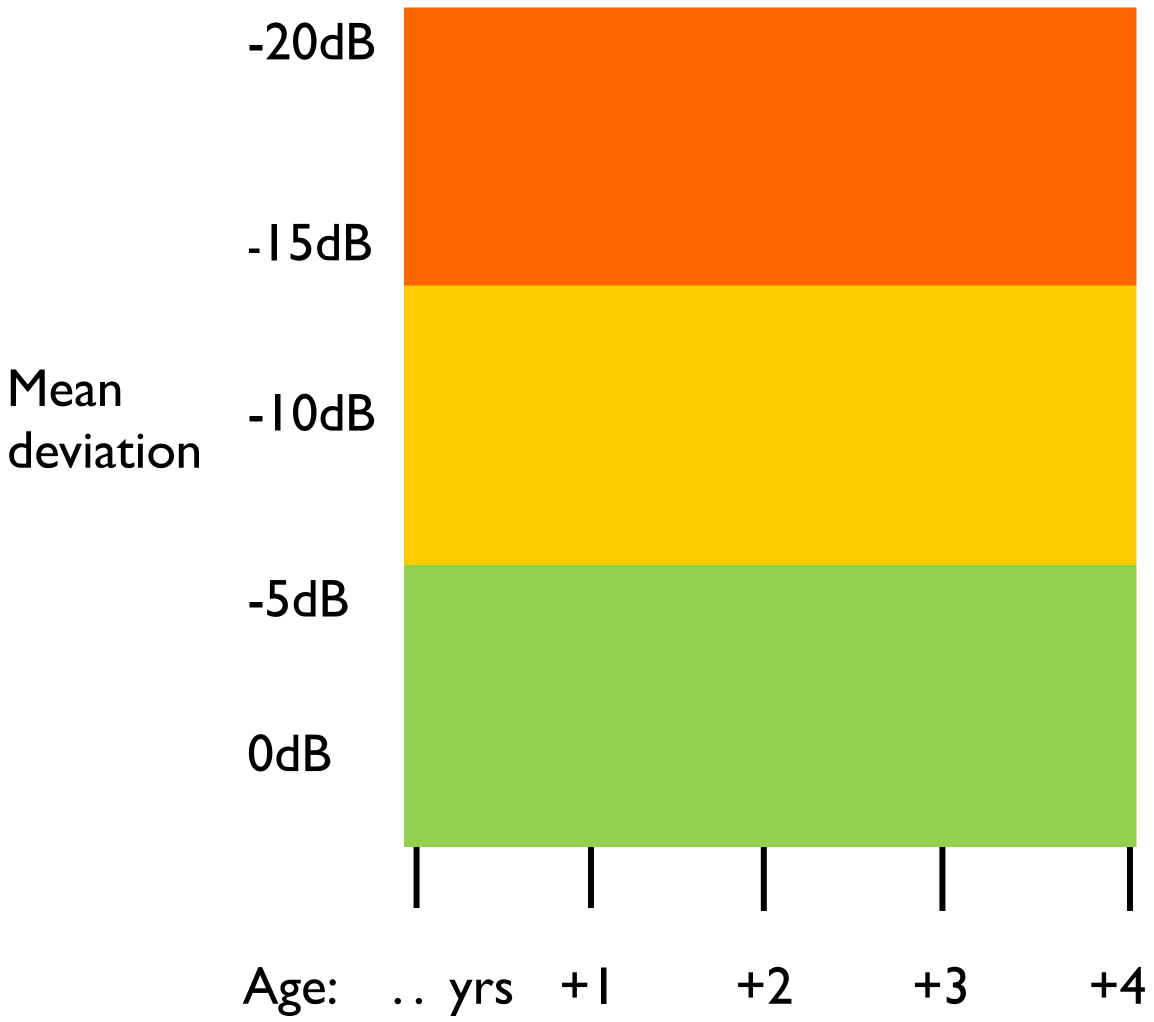

Your eye pressure, Right Eye

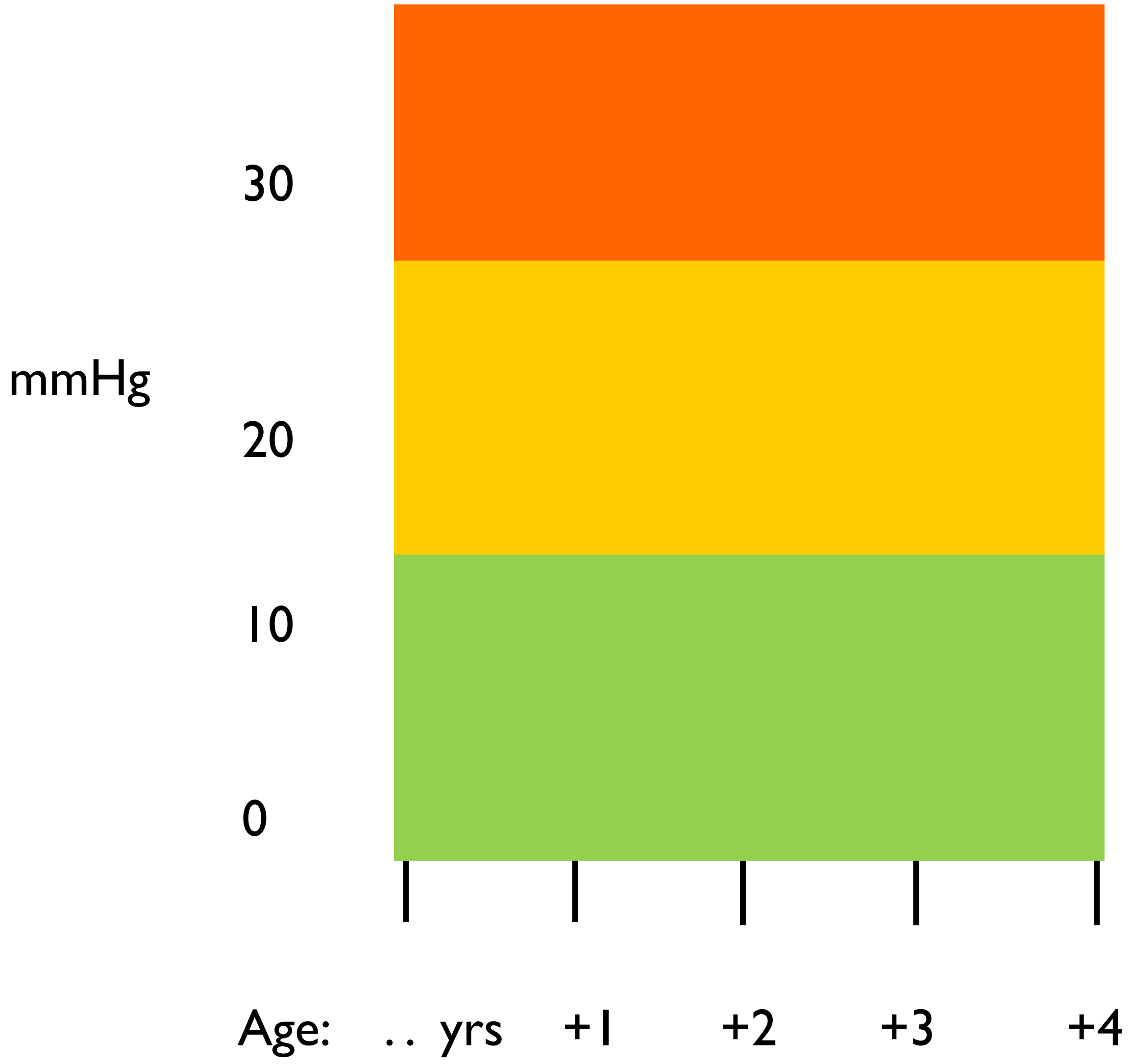

Date (dd/mm/yyyy)	Target pressure (mmHg) for Right Eye

Your eye pressure, Left Eye

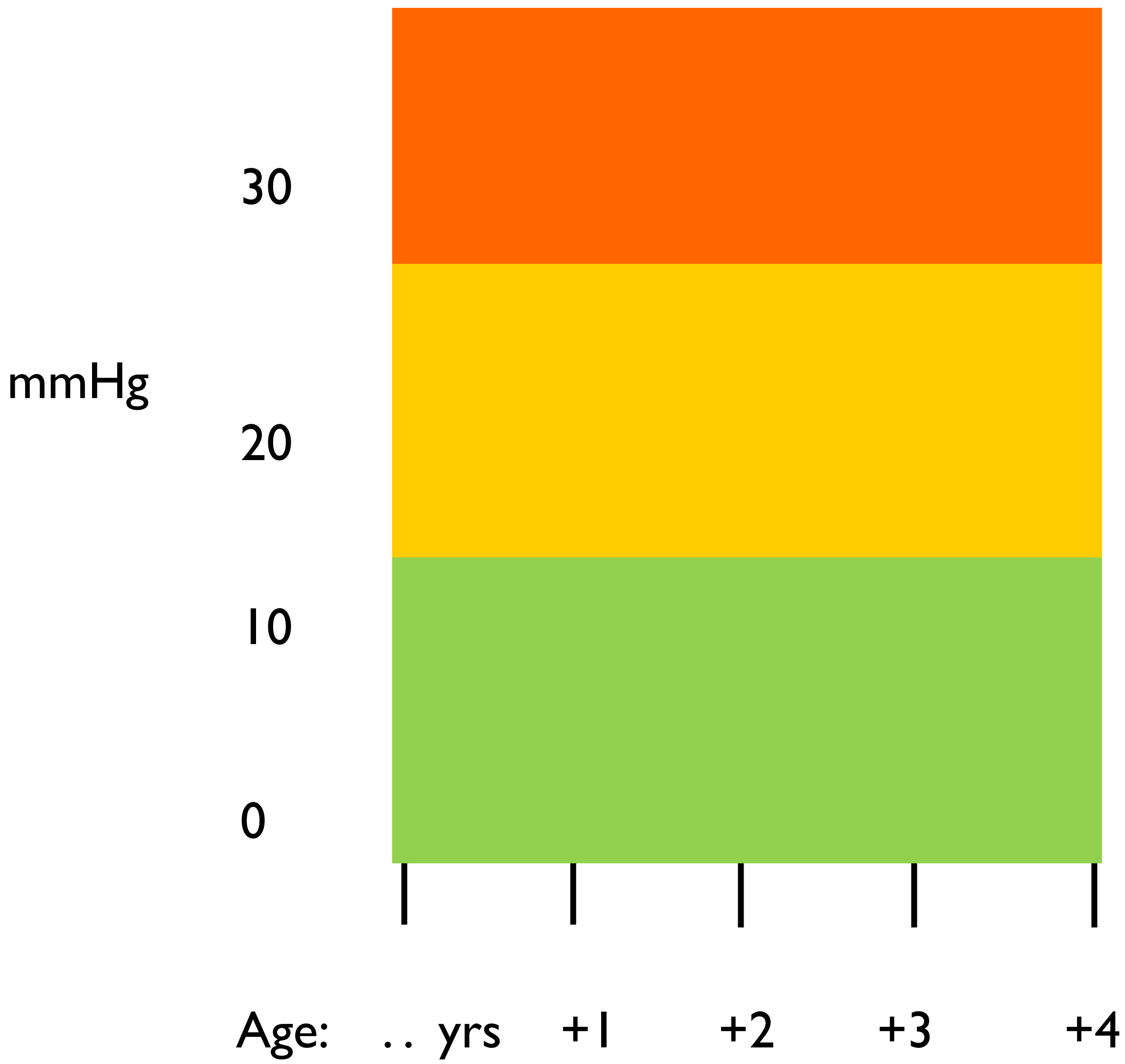

Date (dd/mm/yyyy)	Target pressure (mmHg), for Left Eye

Plan of your care

Appointment : (dd/mm/yyyy)		
	Right Eye	Left Eye
Eye drops prescribed:<br><br>Date eye drops started / ongoing	Yes / no<br><br>……………<br>dd/mm/yyyy	Yes / no<br><br>………………..<br>dd/mm/yyyy
Eye Drops: Name		
Dose / Frequency		
Notes:		
List of other medication:		

Plan of your care, continued

Appointment : (dd/mm/yyyy)		
	Right Eye	Left Eye
Eye drops prescribed:<br><br>Date eye drops start-ed / ongoing	Yes / no<br><br>……………<br>dd/mm/yyyy	Yes / no<br><br>………………..<br>dd/mm/yyyy
Eye Drops: Name		
Dose / Frequency		
Notes:		
List of other medication:		

Plan of your care, continued

Appointment : (dd/mm/yyyy)		
	Right Eye	Left Eye
Eye drops prescribed:<br><br>Date eye drops start-ed / ongoing	Yes / no<br><br>……………<br>dd/mm/yyyy	Yes / no<br><br>………………..<br>dd/mm/yyyy
Eye Drops: Name		
Dose / Frequency		
Notes:		
List of other medication:		

Patient Record of appointments with other **eye** healthcare staff

This page can be used by you/your carer/relative to keep track of when you saw a healthcare professional for your eyes. Any eye-related appointment in community or hospital can be added. Hospital ophthalmology staff will use the table to enter dates of laser treatment and/or surgery

Date	Seen by	Measures taken / Actions

Patient Record of appointments with other **eye** healthcare staff, continued

Date	Seen by	Measures taken / Actions
Below: record of ophthalmology (laser)treatments and surgery		
Date	Seen by	Measures taken / Actions

Space for any personal notes or questions you may have for the ophthalmology team

Example question: how can laser treatment help treat my glaucoma?

We try to answer all your questions and concerns during your consultation in the hospital, but you can write questions down here as an *aide memoire*.

Space for any personal notes or questions, continued
